# A rare case of recurrent hydatid cyst of the spleen: A case report

**DOI:** 10.1016/j.radcr.2023.06.069

**Published:** 2023-07-22

**Authors:** Amna Hassan, Aima Azhar, Saad Mazhar, Shreelal Yadav, Mustafa Bin Tahir, Rojan Basnet, Pratik Bhattarai

**Affiliations:** a Combined Military Hospital (CMH), Lahore Medical College & Institute of Dentistry, Lahore, Pakistan; b Fatima Jinnah Medical University, Lahore, Pakistan; c King Edward Medical University, Lahore, Pakistan; d Manipal College of Medical Sciences, Pokhara, Nepal; e Army Medical College, Rawalpindi, Pakistan

**Keywords:** Echinococcosis, Hydatid cyst, Splenectomy

## Abstract

Cystic echinococcosis (CE), or hydatid disease, is a parasitic infection caused by *Echinococcus granulosus* endemic to areas with considerable pastoral farming and animal husbandry. Typical presentations include hydatid cyst formation in the liver, lungs, brain, kidneys, or bones. An isolated splenic hydatid cyst is an extremely rare occurrence, accounting for only 0.5%-4% worldwide incidence rates, and recurrent cases are even more infrequent. Globalization, cross-border travel, and altered immigration patterns over time have shifted some of the burden of CE from the developing to the developed world, making the diagnosis challenging for these nonendemic areas. Judicious use of imaging modalities for prompt diagnosis and effective intervention is necessary to treat the initial disease and prevent a recurrence. Herein, we present the case of a 13-year-old male with recurrent isolated splenic hydatid cyst. The patient presented with chronic and nonradiating pain in his left hypochondrium. Physical examination revealed splenomegaly. Ultrasonography showed multiple cysts. Computerized tomography (CT) scan showed cystic lesions in splenic parenchyma with numerous internal enhancing septae. Surgical evacuation was performed for the management of disease.

## Introduction

Cystic echinococcosis (CE), or hydatid disease, is a parasitic infection caused by *Echinococcus granulosus*. Sheepdogs are the definitive hosts, while sheep, goats, cattle, and pigs are intermediate hosts [1]. While most cases in humans are asymptomatic, CE typically causes slow, progressively growing cysts in the liver, followed by the lungs, kidneys, bones, and brain. The involvement of the spleen, pancreas, and heart is rare, and reports of isolated splenic infections are even more rare [2,3]. Only 4% of abdominal hydatid disease cases are splenic hydatid diseases [4]. According to the World Health Organization (WHO), about one million people worldwide are infected with CE at any time [5]. There is a high disease incidence in regions with pastoral farming and animal husbandry. Pakistan is endemic to CE, 1 of the 18 neglected tropical diseases, according to WHO [6,7]. The best approach to diagnose is computerized tomography (CT) scan used in conjunction with serological tests for detection of echinococcal antigens in blood. Treatment is based on the size and number of cysts and may include drugs like albendazole, percutaneous treatment, and surgery [8].

The rarity of splenic hydatid cysts and the sparsity of available literature poses a diagnostic challenge for endemic and nonendemic countries alike. It may cause disease progression to life-threatening complications like anaphylactic shock [9]. Herein, we report the case of a 13-year-old male child with a recurrent isolated splenic hydatid cyst.

## Case presentation

A 13-year-old male presented to the emergency department of hospital with a compliant of abdominal pain. The patient reported chronic and nonradiating pain in his left hypochondrium which was progressive for over a year. Splenomegaly was evident on physical examination. His family history was unremarkable.

**Fig. 1:**
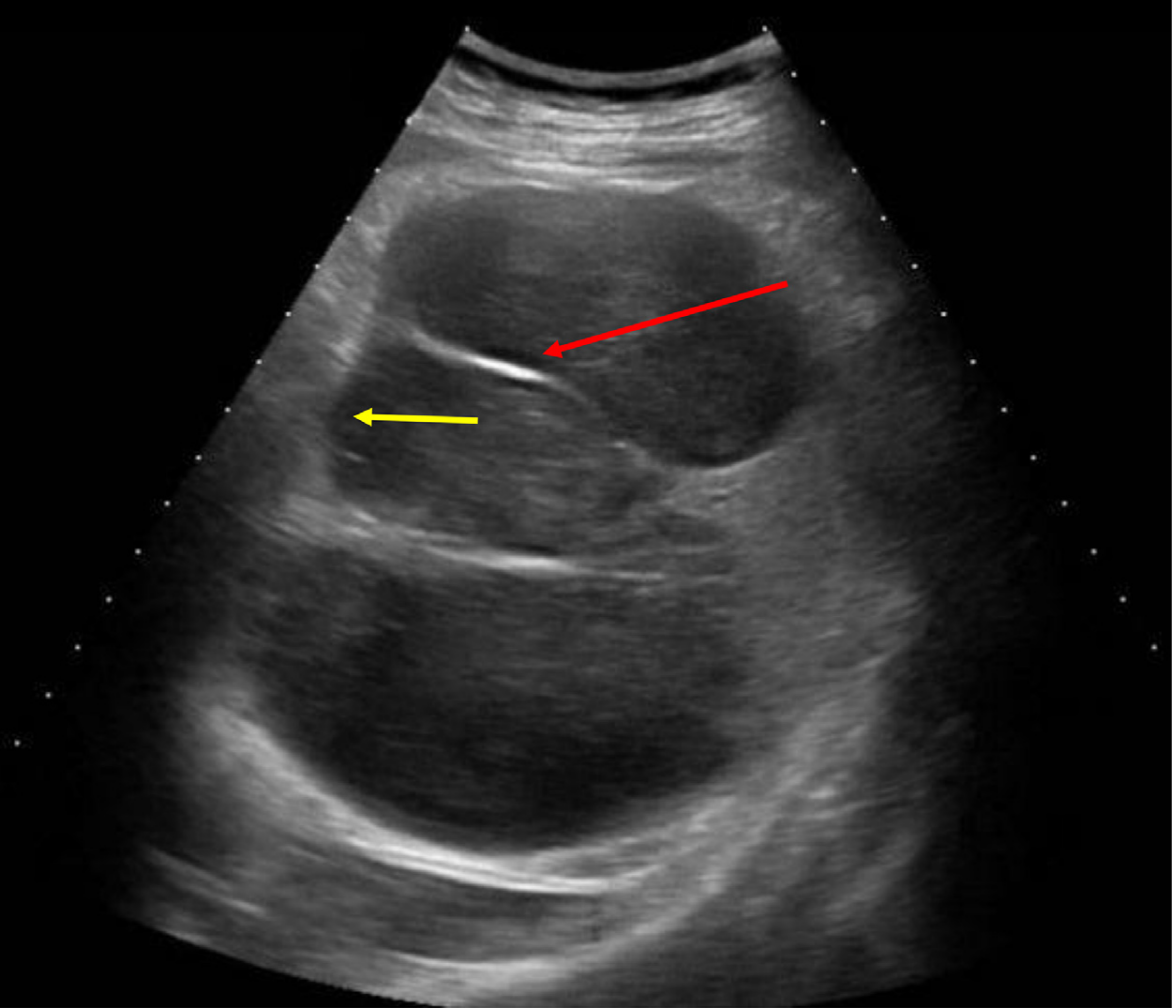
showing septa within cyst (red arrow) and cystic membrane (yellow arrow).

**Fig. 2:**
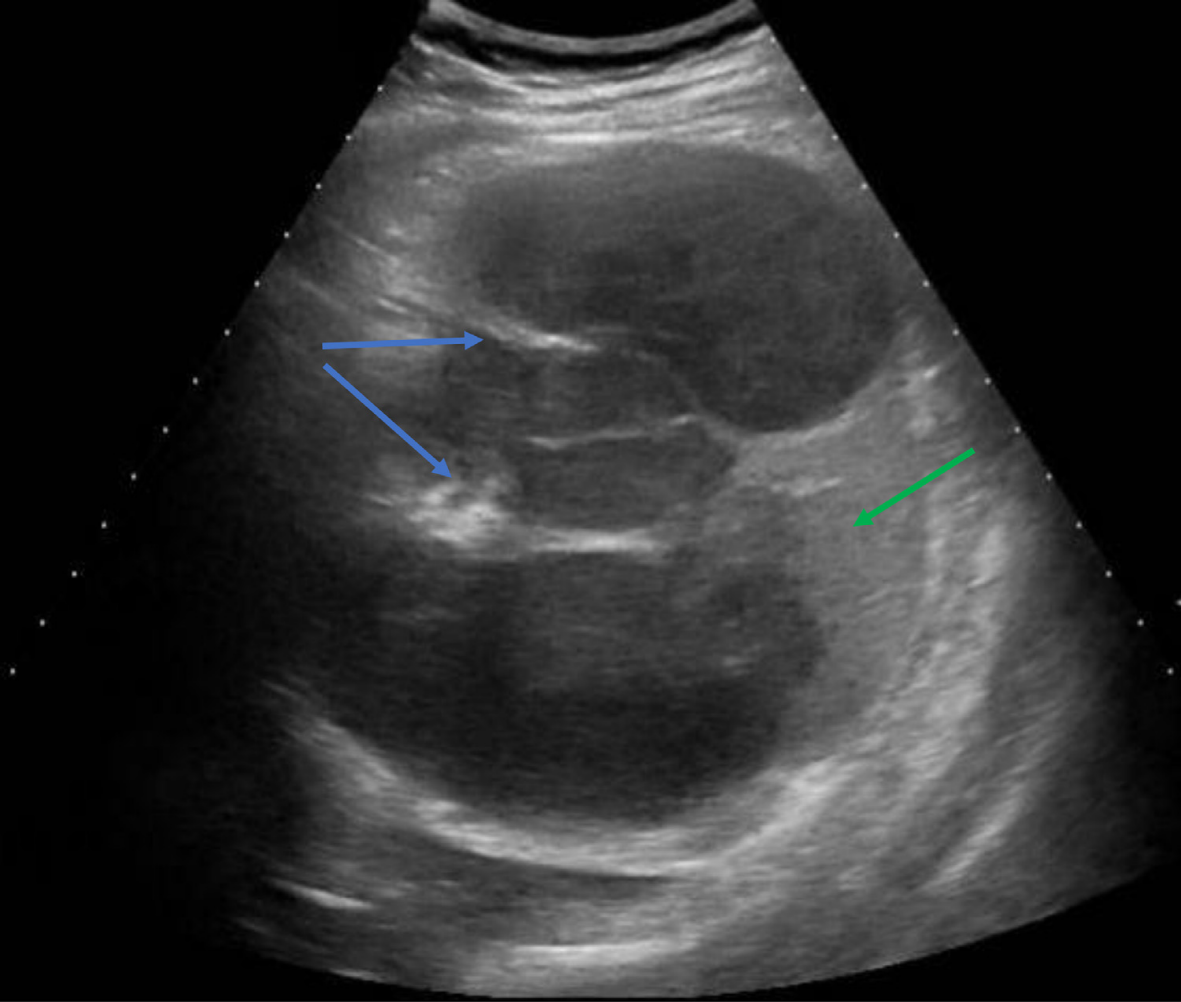
showing multiple septa (blue arrows) and normal spleen (green arrow).

All the routine laboratory tests of the patient were unremarkable. Ultrasonography (USG) of the abdomen was done which showed multiple cysts in the spleen. The patient was then referred to the pediatric surgery department where he was kept under observation. A CT scan was advised which revealed multiple cystic lesions in splenic parenchyma inferiorly displacing the left kidney along with numerous internal enhancing septae. The ultrasonography features are shown in Figures 1 and 2. Histopathologic examination revealed laminated cyst wall encircling many scolices with a double layer of hooklets; consistent with *Echinococcus granulosus* infection thus confirmed the diagnosis of splenic hydatid cyst. The patient was then scheduled for a cystectomy and partial splenectomy. Although the surgery was successful, USG revealed recurrence during the early postoperative follow-up period. The recurrence was managed through surgical evacuation of the splenic cysts.

The differential diagnoses of splenic hydatid cyst include pseudocysts, epidermoid cysts, splenic abscesses, cystic neoplasms, and hematomas of spleen.

## Discussion

Hydatid disease is a serious public health concern, particularly in cattle and sheep-raring countries like Pakistan, the Middle East, South America, Australia, and North Africa, where the disease is endemic. The spleen serves as the site of 4 main types of cysts; epidermoid cysts, post-traumatic pseudocysts, intrasplenic pancreatic pseudocysts, and hydatid cysts due to *Echinococcus granulosus* infections [10]. Among these, an isolated splenic hydatid cyst is an extremely rare manifestation, even in endemic countries, with a worldwide incidence rate of 0.5%-4% [11]. Hydatid disease primarily affects the liver and the lungs, and the involvement of viscera like the kidneys, pancreas, and spleen is infrequent [3]. The rarity of splenic infection results from the fact that most cyst embryos become entrapped within the liver and lungs, and only around 15% manage to enter into systemic circulation [12].

Clinical presentation is variable and depends on the number and size of the cysts. Splenic hydatid cysts are predominantly asymptomatic; however, compression of adjacent viscera through an increase in the size of the cyst causes pain, usually in the right hypochondrium. The patient may also present with a rupture of the splenic cyst and spread of infection to other organs or systemic hypertension resulting from renal artery compression [4]. This aggressive behavior of an isolated splenic hydatid cyst increases the risk of patient mortality. Hence, judicious use of imaging modalities is necessary for prompt diagnosis and effective intervention [13].

The appearance of hydatid cysts on radiographic examination is variable and dependent on factors like location and age. Moreover, complications like infection and rupture make diagnosis difficult. Splenic hydatid cyst poses a diagnostic challenge, particularly in nonendemic areas, owing to shared similarities with other cystic lesions of the spleen on sonography and CT scan. It should be included in the differential diagnosis of any cystic lesion of the spleen, especially in endemic areas [9,12]. CT scan remains the investigation of choice, and it is used in conjunction with immunological tests to solve the challenge of diagnosis. CT scan also guides treatment and ensures complete surgical resection of the cyst. Splenectomy is the conventional treatment, with the choice of either total or partial splenectomy depending upon the degree of involvement of splenic parenchyma [14]. Albendazole is given ad adjunctive treatment, reducing the recurrence risk. However, microscopic spillage of parasites, incomplete surgical removal of all viable cysts due to inaccessibility, and cystic residues at the site of the initial operation are postulated as the reason behind isolated recurrent splenic cysts managed surgically. Five to 10% of these surgical cases are associated with spillage of protoscolex-rich fluid. The recurrence is treated conservatively through surgical removal with or without partial splenectomy [9].

The increasing globalization, cross-border travel, and changing immigration patterns have shifted some of the disease burden from the developed to the developing world, making isolated splenic hydatid cyst a global health challenge. Moreover, the disease is difficult to diagnose and a delay in treatment leads to serious life-threatening complications. The sparsity of available literature further adds to the challenge, making adequate diagnosis and intervention for isolated splenic hydatid cysts a persistent problem for endemic and nonendemic areas alike.

## Conclusion

An isolated splenic hydatid cyst is an extremely rare occurrence, with a worldwide incidence rate of 0.5%-4%. The appearance of hydatid cysts on radiographic examination is variable and dependent on factors like location and age. Moreover, complications like infection and rupture make diagnosis difficult. The difficulty of diagnosis and scarcity of available literature make prompt, effective diagnosis and intervention challenging. CT scan is the diagnostic modality of choice and aids treatment. This unique case highlights the importance of proper preoperative imaging diagnosis, which guides the choice of treatment and reduces the risk of disease complications and recurrence.

## Ethical approval

Not required as we have acquired consent from the patient.

## Research registration

1. Name of the registry: NA
2. Unique Identifying number or registration ID: NA
3. Hyperlink to your specific registration (must be publicly accessible and will be checked): NA

## Provenance and peer review

Not commissioned, externally peer reviewed.

## Author contribution

All authors contributed equally.

## Patient consent

Written informed consent was obtained from the patient for publication of this case report and accompanying images. A copy of the written consent is available for review by the Editor-in-Chief of this journal on request.
